# Chemical Recycling of High-Molecular-Weight Organosilicon Compounds in Supercritical Fluids

**DOI:** 10.3390/polym14235170

**Published:** 2022-11-28

**Authors:** Igor V. Elmanovich, Victor E. Sizov, Vadim V. Zefirov, Alexandra A. Kalinina, Marat O. Gallyamov, Vladimir S. Papkov, Aziz M. Muzafarov

**Affiliations:** 1 A.N. Nesmeyanov Institute of Organoelement Compounds, Russian Academy of Sciences, Vavilova 28, 119991 Moscow, Russia; 2 Faculty of Physics, Lomonosov Moscow State University, Leninskie Gory 1-2, 119991 Moscow, Russia; 3 Enikolopov Institute of Synthetic Polymer Materials, Russian Academy of Sciences, Profsoyuznaya 70, 117393 Moscow, Russia

**Keywords:** polydimethylsiloxane, silicone rubber, supercritical fluids, recycling, depolymerization, destruction

## Abstract

The main known patterns of thermal and/or catalytic destruction of high-molecular-weight organosilicon compounds are considered from the viewpoint of the prospects for processing their wastes. The advantages of using supercritical fluids in plastic recycling are outlined. They are related to a high diffusion rate, efficient extraction of degradation products, the dependence of solvent properties on pressure and temperature, etc. A promising area for further research is described concerning the application of supercritical fluids for processing the wastes of organosilicon macromolecular compounds.

## 1. Introduction

The accumulation of huge amounts of plastic waste is one of the global challenges that the world is currently facing. Indeed, the serious environmental risks of landfills or the incineration of plastic waste are a growing global concern. Under these conditions, the inevitability of the transition of the polymer industry in the foreseeable future towards the production of either biodegradable or easily recyclable polymers is obvious. Therefore, the biodegradable or easily recyclable polymers can be called the “polymers of the future”. However, in the case of biodegradable materials, one may fear deterioration of consumer properties as a result of preliminary storage for too long or the artificial induction of premature degradation. On the other hand, chemical recycling seems promising, allowing the plastic waste to be disassembled into initial monomers or other useful compounds. The search for new recycling strategies is constantly intensified. The polymers to be disassembled to small building blocks are conceptually reminiscent of Kiddicraft or Lego building bricks: after the end of the usage of the product, it is subject to disassembly into elementary bricks, which, after a simple cleaning procedure for monomers, are again used to build new products.

Unlike most commonly used organic polymers, high-molecular-weight organosilicon compounds (polysiloxanes, silicones) fit well with this strategy. Indeed, not only can they be obtained using waste-free green technologies, but they can also be recycled to initial monomers, oligomers, or derivatives. The present review addresses the specific issue of chemical recycling of polysiloxanes.

In particular, currently there are several strategies for solving the increasingly pressing problem of efficient disposal of plastic waste: (1) mechanical recycling, (2) disposal in specially equipped landfills, (3) incineration with energy release, and (4) chemical recycling. Mechanical recycling ideally does not lead to changes in the chemical structure of the polymer (or these changes are minor) and is certainly an attractive method for the recycling of plastics. However, the widespread use of the mechanical recycling of polymers is fundamentally limited due to high requirements for the purity of feedstock materials, the accumulation of defects in the chemical structure of macromolecules, and the gradual decrease in performance with each processing cycle. Concerning environmental risks, as compared to hydrocarbon polymers, polysiloxanes seem to be relatively safe for landfilling since they are bioinert and readily decompose in soil into hydroxyl derivatives of silicon and low-molecular-weight organosilicon oligomers [1,2,3]. However, the consumption of silicones is growing rapidly, since they are widely used in cosmetics, pharmaceuticals, personal care products, cleaning products, and other everyday goods. Moreover, the degradation products of polysiloxanes are volatile [4], and their possible impact on the environment and living organisms is still not fully understood (indeed, even inert inorganic silica in a highly dispersed state is not completely safe) [5,6,7,8]. All the mentioned factors are of growing concern to the scientific community and promote the search for new strategies for the recycling of polysiloxanes.

It should also be taken into account that the reduction of silicon from silica feedstock and the production of silicon-containing monomers for the subsequent synthesis of polysiloxanes is a very complex and economically (energetically) costly process, accompanied, moreover, by environmentally unfavorable emissions. This situation differs from the production of plastics from fossil gas. The landfilling of silicon already activated for chemical transformations and its subsequent conversion again into the oxidized form of silica seems to be an undesirable waste of resources.

This economic consideration fully applies to the use of waste polysiloxanes as raw materials for waste incineration plants. Indeed, during the incineration of polysiloxanes, dispersed silica can be formed. Although the obtained material can potentially be in demand in chemical technology (for example, as a filler for polymer-inorganic composites), it would not be economically viable to use it for the further production of polysiloxanes due to chemical inertness. Thus, for polysiloxanes, to an even greater extent than for hydrocarbon polymers, it is highly desirable to develop chemical processing, in which changes in the chemical structure of the polymer lead to the appearance of new low-molecular-weight products valuable for the chemical industry, in particular, suitable for subsequent re-synthesis of polysiloxanes.

The chemical recycling for polysiloxanes is not only more feasible from an economic point of view than for hydrocarbon-based plastics but also has a well theoretically and experimentally developed universal strategy: the process of catalytic and/or thermal destruction of polysiloxanes with the formation of low-molecular-weight cyclic siloxanes, which can act as monomers for the new synthesis of organosilicon polymers. The catalytic variant of this process can take place at relatively low temperatures, up to 250 °C, and when using reactive nucleophilic or electrophilic catalysts, even at lower temperatures, down to room temperature. Currently, factories for the processing of silicone rubber and silicone fluids are emerging and expanding their production. On the other hand, the governments of a number of countries are taking steps to include lower siloxane cycles in the lists of substances hazardous to humans and the environment. It is highly probable that with the growth in silicone production, combined with the persistent trace presence of siloxane cycles in the silicone industry products, as well as the government incentives for polymer waste disposal and tightening emission control regulations, we will see a wider industrial implementation of the described chemical recycling process for polysiloxanes. At the moment, the main industrial capacities for the recycling of silicones are concentrated, logically, in China [9], which is also the main producer of silicones in the world. The participants of the international conference ISOS-2017 in Jinan were given the opportunity to visit the existing production of silicone sealants from silicone rubber waste in Baolong, China. The enterprise exists and is successfully developing to this day, which can be considered as confirmation of the economic efficiency of silicone waste processing.

Among the disadvantages inherent in any technological process, one can note the high energy consumption during the thermal catalytic method of decomposition of polysiloxanes and the formation of gaseous impurities that need to be captured and disposed of. There are very few of them, but the threshold limit values for any organics are quite strict, so the complications associated with the additional purification of the product are inevitable.

Therefore, research aimed at finding new low-cost close-loop recycling processes is of particular relevance, and this applies both to silicones and to other mass-produced polymeric materials. One of the promising directions of this scientific research seems to be the use of supercritical fluids for the recycling of polysiloxanes. The goal of this review is to point out a possible promising “overlap” between the fields of chemical recycling of silicones and the application of supercritical fluids in the recycling of plastic waste.

## 2. General Approaches to the Chemical Recycling of Polysiloxanes

### 2.1. Thermal Decomposition of Siloxanes

Thermal decomposition of polysiloxanes at temperatures of 400–650 °C is the most studied process among all approaches [10,11,12]. The thermal decomposition occurs in a vacuum or in an inert atmosphere with the formation of cyclic oligomers at these temperatures. The presence of oxygen leads to a significant decrease in the required temperature: down to ≈290 °C. The most common reaction product of the thermal decomposition of silicones is cyclic trimers; in this case, tetramers, pentamers, and larger cyclic oligomers are formed in smaller amounts [12].

**Figure 1:**
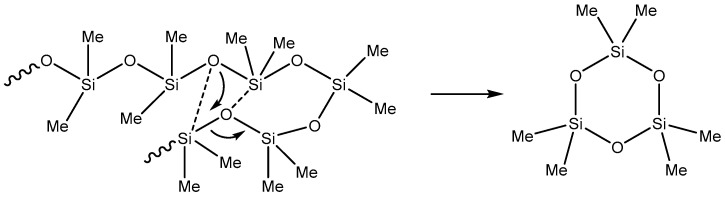
The mechanism of formation of hexamethyltricyclosiloxane. Adapted from Ref. [11], copyright 2001, with permission from Elsevier.

Thermal decomposition of PDMS can proceed through two competing mechanisms, depending on the heating rate and temperature. At slow heating rates, decomposition is determined by molecular structure and chain flexibility. The weakest PDMS bond is the C–Si bond (76 kcal mol^−1^); however, the formation of cyclic oligomers during the decomposition of PDMS suggests the Si–O bond to be primarily broken (108 kcal mol^−1^) [11]. PDMS depolymerization was supposed to be determined mainly by molecular structure and kinetic considerations rather than by binding energies [11]. It is assumed that a rearrangement of the siloxane bond occurs, which leads to the elimination of the cyclic dimethylsiloxane and a reduction in the residual chain length. This mechanism can be illustrated by the formation of the smallest cyclic product, hexamethylcyclotrisiloxane (Figure 1). Such a process continues until the linear chain becomes too short for the cycle formation or the characteristic evaporation times of oligomeric fragments become shorter than their cyclization times.

The mechanism shown in Figure 1 suggests cycles formation from the middle of the polysiloxane chain, which requires sufficiently high temperatures (more than 500 °C). At lower temperatures, the degradation process is preferably initiated closer to the end groups as far as the end groups or impurities activate the degradation mechanism.

Even silanole end groups, as well as impurities, silanolate end groups, or acid groups can act as active centers for depolymerization. At temperatures above 300 °C, the terminal silanol groups are the Achilles’ heel of siloxane macromolecules responsible for the heterolytic decomposition of the siloxane bond by the “biting off” mechanism of the chain fragments cyclizing. Indeed, the replacement of terminal silanol groups with trimethylsiloxy groups makes it possible to increase temperature stability of PDMS up to 500 °C [13,14,15].

Usually, a very high equilibrium content of low-molecular-weight cyclic products in linear polysiloxanes is noted, which make up significant fractions even at low temperatures [13,14,15]. This implies their further growth with increasing temperature. Therefore, the efficient extraction of low-molecular-weight degradation products should shift the equilibrium towards depolymerization and promote this process.

When heated rapidly to 800 °C, the degree of polymerization of decomposition products moves towards the region of longer cyclic oligomers [16]. Thus, the most frequent product is a tetramer, rather than a trimer, as during slow heating to 400 °C. Another reaction product in the case of rapid heating is methane, which is released as a result of the cleavage of the Si–CH_3_ bond and hydrogen elimination.

Other heat-resistant phases such as silica can also be formed during decomposition. According to the results of TGA, the mass of silica can reach 50% at low heating rates and 10% at high heating rates in oxygen atmosphere [11]. Such a sharp drop is due to the fact that with an increase in heating rate, chemical reactions proceed at higher temperatures, while the solubility of oxygen in the condensed phase decreases, and the rate of thermal destruction reactions rises. Under these conditions, the occurrence of a heterogeneous reaction of active radicals with atmospheric oxygen becomes less probable.

Thermal oxidation of PDMS occurs due to the oxidation of methyl groups and represents a chain destruction reaction. At temperatures up to 200 °C, the low rate of oxidation is caused by an unusually slow (compared to hydrocarbons) initiation reaction. At higher temperatures, additional chain initiation mechanisms appear. The replacement of methyl groups by phenyl ones enhances the stability of macromolecules up to 250 °C, which is explained not only by the greater oxidative stability of the latter but by their inhibitory effect on the oxidation of the former as well. Impurities containing transition metals can serve as antioxidants that inhibit the thermal oxidation of siloxane chains as a whole [17,18,19].

The results of the decomposition kinetics analysis and calculation of the activation energies of organosilicon polymers, including polydimethylsiloxanes, were described in Ref. [20]. According to this study, one of the main disadvantages of the method of thermal decomposition of siloxanes is the relatively slow kinetics.

### 2.2. Catalytic Decomposition of Siloxanes

**Figure 2:**
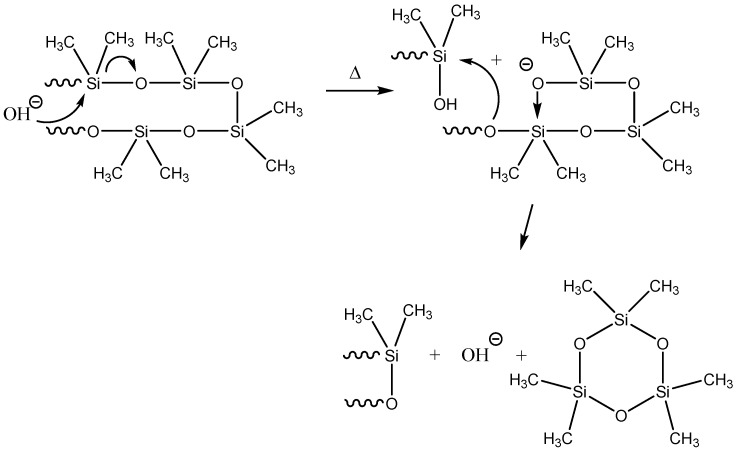
Process of thermal decomposition of PDMS in the presence of KOH. Adapted from Ref. [21] by permission from Springer, copyright 2000.

One of the pioneering studies of the catalytic decomposition of PDMS was the Ref. [10], in which it was shown that the presence of the KOH impurity (used to catalyze the polymerization of PDMS itself) accelerates the process of PDMS thermal decomposition. In this case, the rupture of the Si–O bond occurs due to nucleophilic substitution with the participation of hydroxyl ions (Figure 2) [21].

In turn, Si–O bond cleavage can be promoted using both nucleophilic and electrophilic reagents and/or catalysts; thus, in order to reduce the energy and time costs for the decomposition process, various catalytic systems are being actively studied. For example, it was proposed to use inexpensive catalysts based on iron and zinc salts for the Si–O bonds activation in PDMS and their concomitant cleavage using benzoyl fluoride, benzoyl chloride with various substituents, other organic acid chlorides, acetic anhydride, acetic acid, methanol, or fatty alcohols to obtain silicon-containing monomers [22,23,24]. However, these reactions are slow and require high temperatures. The same authors also used boron trifluoride diethyl etherate (BF_3_OEt_2_) to depolymerize PDMS at lower temperatures (100 °C) [25,26]. The process was complicated by the formation of boron-containing by-products requiring regeneration, yet under optimized conditions in the absence of solvents the monomer yield was rather high.

In turn, it was shown in Ref. [27] that the deposition of PDMS on highly dispersed Fe_2_O_3_/SiO_2_ composite substrates catalyzes the thermal decomposition of PDMS and increases the intensity of depolymerization processes. A similar effect was achieved when PDMS was deposited on phosphorus-containing silica [28]. Thus, the deposition of PDMS onto appropriate substrates can serve as a tool for lowering the decomposition temperature.

**Figure 3:**
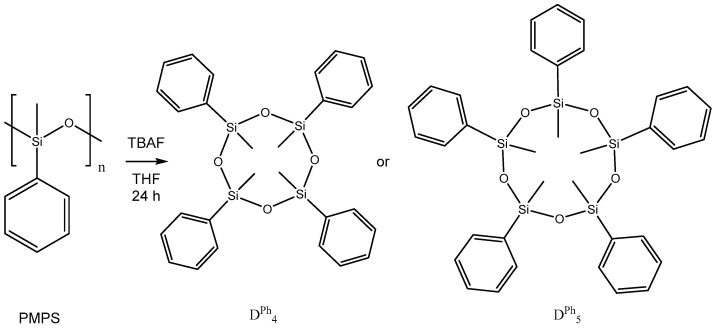
Fluorine-ion-catalyzed depolymerization of polyphenylmethylsiloxane. Adapted with permission from Ref. [29], copyright 2021, American Chemical Society.

Recently, a group of authors proposed a method for the low-temperature decomposition of polysiloxanes (PDMS and PMPS) in the presence of catalytic amounts of tetrabutylammonium fluoride in some organic solvents, among which the best results were obtained with THF (Figure 3) [29]. The authors demonstrated the effectiveness of the selected catalyst both on model compounds and on commercially available silicone fluids and elastomers. The decomposition products were mainly cyclic siloxanes D_4_, D_5_, D_6_. The work shows that the resulting cyclic siloxanes can be re-polymerized, which makes the proposed process promising from an industrial point of view.

**Figure 4:**
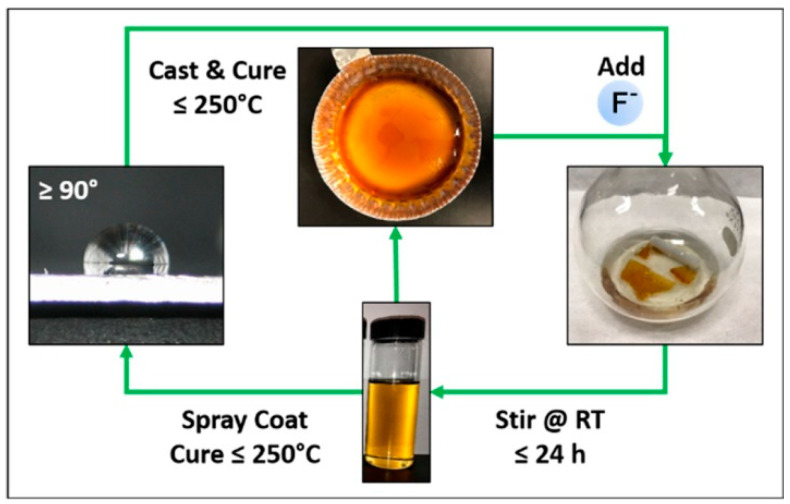
Recycling cycle for crosslinked silicone rubbers proposed in Ref. [30]. Reproduced from Ref. [30], copyright 2019, American Chemical Society. Further permission related to the material excerpted should be directed to the ACS.

The processes of catalytic depolymerization of polysiloxanes are of particular interest for crosslinked systems formed in the presence of tri- and tetrafunctional silanes, which form the corresponding crosslinks. Such thermosetting silicone rubbers have improved heat resistance and other advantageous properties, but are particularly difficult to recycle. In this case, reversible de-linking, re-molding, and re-crosslinking appears to be the ideal strategy for recycling highly crosslinked silicone rubbers. An example of such a process was given in Ref. [30]: in this work, silicone rubber was dissolved in THF containing fluorine ions (tetrabutylammonium fluoride, TBAF, served as the source of F^−^). The presence of fluoride ions in THF leads to the complete dissolution of silicone rubber in this system. The resulting solution was applied to the surface by spraying, after which it was subjected to heat treatment, which led, after removal of the solvent, to the recovery of the polymer network and the formation of a superhydrophobic crosslinked silicone coating (Figure 4).

Another original approach to PDMS depolymerization is the “natural” one using soils or bacteria. In a number of works, the decomposition of PDMS in soil was studied [31,32] since this is the most important environmental aspect for any polymer. PDMS was shown to be unstable in soil media under the catalytic action of clays, although specific degradation mechanisms were not described. In Ref. [33], a comparative study of the catalytic effect of clay minerals of various nature on the PDMS degradation was carried out. Kaolinite, beidellite, and nontronite were the most active ones. It was noted in a number of publications that some bacteria are able to promote the further biodegradation of low-molecular-weight organic degradation products of PDMS in the soil; however, the rates of such processes are low, and the results described in the literature are not always reproducible [34]. It can also be noted that the Si–C bond does not occur in nature, and, as a result, there are no enzymes for its cleavage, although other cleavage mechanisms are possible in metabolism, including those of higher organisms [34].

To date, many different systems have been proposed to optimize the depolymerization and recycling of silicones. We refer interested readers to a recently published Ref. [35], where the authors provide a detailed analytical review of studies on the degradation of polysiloxanes in relation to their recycling. The authors analyze in more detail than in this review the options for carrying out the destruction of polysiloxanes, including different temperature ranges and using various catalytic systems. Particular attention is paid to the mechanisms of these processes. In conclusion, the authors emphasize that the bulk of research in the field of thermal degradation of polysiloxanes is focused on improving the understanding of the chemistry of the process but not on the development of recycling methods themselves. As rare examples of works in which the process of thermal degradation of polysiloxanes is accompanied by the subsequent use of low-molecular-weight (or non-crosslinked) products for the further synthesis of new polysiloxanes, the authors of the Ref. [35] refer readers to publications [23,29,30].

There are still a few examples of commercial implementations of chemical recycling processes for siloxanes, including silicone fluids and silicone rubber, which are described in detail. One of the products of such utilization of siloxane waste is re-polymerized high-molecular-weight PDMS [36].

In addition to crosslinking agents, siloxanes are sometimes modified with various fillers. For example, silicone rubber contains fillers (mainly aerosil), which radically improve the complex of its physical and chemical properties. The properties of the filler also affect the course of depolymerization processes. When recycling such composite rubber, it becomes necessary to efficiently extract and then reuse fillers as well, which increases energy costs and complicates the technology. In the industrial processes mentioned above, extracted fillers are used as additives in the production of cements.

The increase in plastic waste, including siloxanes, is a global problem. Attempts are being made to improve the technology for the thermal recycling of siloxanes, but only a few processes are about to enter the real market. In this regard, the search for new ideas and approaches to the recycling of polysiloxanes remains a demanding task. One such idea could be the use of supercritical (sc) fluids. Supercritical fluids are dense but highly mobile fluids. As a result, the processes of extraction, dispersion, separation, dissolution, and swelling are significantly accelerated in them. That is why the possible synergistic optimization of processes using supercritical fluids for depolymerization, both thermal and catalytic, is of interest. There are several examples of patenting depolymerization processes in sc media [37]. Against the background of tightening regulation in the production and recycling of siloxanes [38], an important positive factor is the environmental friendliness of typically used supercritical fluids.

Thus, environmental friendliness, the high diffusion rate of supercritical fluids, the pronounced swelling of the polymer in them, the efficient extraction of low-molecular-weight organosilicon products of depolymerization (which leads to an effective shift in the equilibrium of the system towards low-molecular-weight reaction products), the facilitating of the dispersion of inorganic degradation products and residual filler, and the ease of separation process fractions by pressure variation are the factors that can speed up reactions, lower the process temperature, and simplify the choice of an easily regenerated catalyst, opening up prospects for the introduction of industrial processes for the utilization of high-molecular-weight organosilicon compounds using supercritical fluids.

## 3. Recycling of Polysiloxanes in Supercritical Media

### 3.1. Plastic Waste Recycling in Supercritical Media

There are hundreds of original research papers and a significant number of reviews that are dedicated to the topic of plastic recycling in supercritical media [39,40,41,42]. Most of the research is focused on the study of polymer degradation processes in sc water (SCW) and sc alcohols; however, there are examples of works in which chemical recycling of plastic is carried out in sc acetone, sc ammonia, and a number of other sc media. As a separate area of research, we can single out the subject of the processing of plastic in sc CO_2_, which we discuss in more detail in the next section. Here we provide a brief description of the main approaches for chemical recycling of the most common plastics in sc water and sc alcohols.

Among all the listed sc fluids, the majority of scientific research is dedicated to the study of plastic recycling in SCW. This is not surprising, given that SCW is extremely reactive, the properties of SCW can be controlled by adjusting solvent pressure or temperature, and SCW is absolutely compatible with the environment. Hydrolysis in sub- and supercritical water of polymers obtained in the polycondensation process, such as poly(ethylene terephthalate) (PET), polyamides, or polycarbonates, can lead to the formation of the corresponding monomers in high yields [43,44,45,46]. The situation is more complicated with the most common vinyl polymers obtained in the polymerization process, e.g., with polyolefins, poly(vinyl chloride) and polystyrene. The thermal destruction of polystyrene and polyolefins in SCW leads to the formation of a multicomponent product composed of solid, liquid, and gaseous phases [47]. Although both oligomers in the solid phase and low-molecular-weight compounds in the liquid and gaseous phases may be of interest to the chemical industry or energy industry, the development of an efficient industrial chemical recycling process in this case is associated with obvious difficulties. It should be noted, however, that the isolation of certain products can be facilitated by separating the liquid reaction product into a polar aqueous phase and a non-polar phase containing aliphatic and aromatic hydrocarbons. A separate niche for the use of SCW can be the chemical recycling of polymers containing chlorine or fluorine atoms, since in this case potentially environmentally hazardous elements are “locked” in the water phase, without harmful emissions into the atmosphere. For example, when poly(vinyl chloride) is recycled in SCW, although the composition of the product turns out to be multi-phase and multi-component, due to the dehydrohalogenation reaction, chlorine atoms quantitatively participate in the formation of hydrochloric acid in the aqueous phase [48]. Similarly, if fluoroplastics are recycled in SCW, fluoride ions are localized in the aqueous phase [49,50]. In Ref. [49], the authors demonstrate that for the process of permanganate-induced thermal decomposition of PVDF and PVDF-based copolymers in SCW, the yield of F^−^ ions in the aqueous phase can reach 100%. For H_2_O_2_-induced decomposition the yield of F^−^ is up to 98%, according to the same authors [50]. For a more detailed analysis of the processes of polymer recycling in SCW we refer readers to the review papers on the matter [39,40,41,42,51].

The motivation for the transition from SCW to sc alcohols is a decrease in operating temperatures and pressures (for comparison, the critical point of water is 374 °C and 217 bar, the critical point of methanol is 240 °C and 78 bar, the critical point of ethanol is 241 °C and 63 bar) as well as reduction of the corrosion load on high-pressure reactor equipment (it is well-known that sub- and sc water in the presence of ions is an extremely corrosive medium). The general trends in the chemical recycling of the common types of plastics remain unchanged during this transition: in the case of recycling of PET, polycarbonates, and polyamides, monomers or chemical derivatives of monomers can be obtained in high yields; for the vinyl polymers the recycling products have complex phase and component composition. The processing of PET in sc methanol is one of the most scientifically and technologically developed processes of this kind. The products of PET methanolysis are dimethyl terephthalate and ethylene glycol, which can be used as monomers for further synthesis of PET [52].

Both mechanical and chemical recycling strategies for PET are currently implemented on industrial level; the obtained recycled PET (rPET) is used in various applications [53]. In relation to the topic of this review, it is important to note that chemical recycling of PET in sc methanol is a rare example of a supercritical fluid recycling process for which industrial implementation has been carried out: a pilot plant for PET methanolysis was launched by Mitsubishi Heavy Industries, Ltd. (Japan).

From a practical point of view, one of the most interesting areas of the application of sc media in the processes of plastic recycling is the chemical recycling of materials of complex composition and structure: crosslinked polymers, resins and vulcanized rubbers [54,55], thermosetting materials [56,57,58,59], carbon-fiber-reinforced polymers (CFRPs), and fiberglass-reinforced plastic materials. For example, the processing of CFRPs in sc water and sc alcohols leads to chemical destruction of the polymer component of the composite while retaining the reinforcing carbon fiber. It is important to note that in this process carbon fiber is not subjected to destruction or deformation, completely retains its mechanical properties, and is ready for reuse. The obvious practical potential of this process explains the presence of a large number of scientific papers in this area; we refer readers to a number of published review articles [60,61,62]. Panasonic Electric Works Co. (Japan) seems to demonstrate interest in the process of processing composite plastics in sub- and sc water, since the company launched a pilot plant that allows the extraction of carbon fiber and other fillers from plastic composites [63].

Thus, the works in which sc water and sc alcohols are used as media for the recycling of plastic waste represent a large independent area of scientific research. These studies cover all the most common plastics as well as composites and crosslinked systems. Among the variety of approaches to plastics recycling in supercritical media, the recycling of silane-crosslinked polyethylene in sc water and sc alcohols is directly related to the topic of this review and we address it in the following separate section.

### 3.2. Recycling of Silane-Crosslinked Polyethylene in SCW and sc Alcohols

Crosslinked polyethylene (XLPE) is a chemically resistant, durable material that can be used, for example, as an insulator for high voltage cables or as a material for water pipes or heating systems. One of the options for chemical crosslinking of polyethylene chains is “silane crosslinking”, in which the crosslinking agent is a silane with various functional chemical groups. Typically, such silanes contain one vinyl group and two or three hydroxyl groups. Then, at the first stage, the silane is grafted to the PE polymer chain (alternatively, ethylene can be copolymerized with vinyl-silane), and at the second stage, the condensation of hydroxyl groups occurs with the formation of siloxane chemical links (–Si–O–Si–) between the PE chains. This process for making silane-crosslinked PE (silane-XLPE) is chemically flexible and economically viable, while the resulting silane-XLPE is a robust high-performance material. At the same time, due to the presence of chemical links between PE chains, this material is particularly difficult for mechanical recycling. The optimal recycling strategy for silane-XLPE seems to be selective de-crosslinking, followed by re-molding and re-setting of the material. Although the siloxane bond in silane-XLPE can undergo selective hydrolysis or alcoholysis, the strong hydrophobicity of silane-XLPE makes it difficult for polar solvents such as water or alcohols to dissolve in the material and participate in the reaction. In this regard, the tunable properties of supercritical fluids, and in particular the decrease in polarity of sc water and sc alcohols with increasing temperature, arguably make them an ideal solvent for the silane-XLPE recycling process.

**Figure 5:**
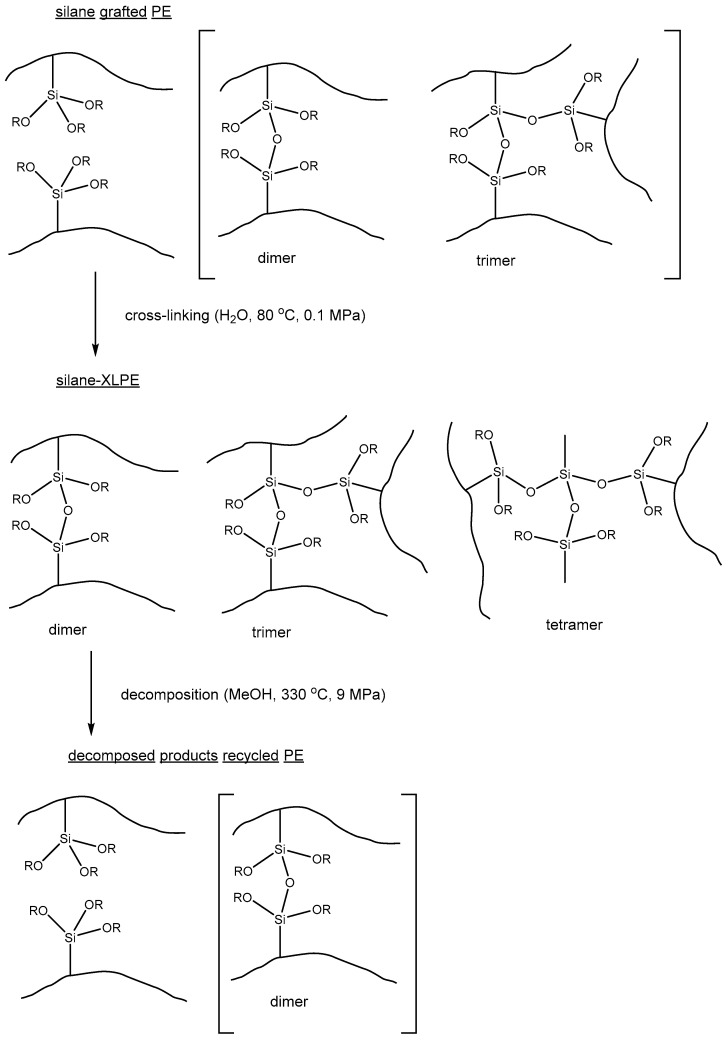
Chemical structures of the silane-XLPE precursor (upper image), silane-XLPE crosslinked product, and selective silane cleavage product (bottom image). Adapted from Ref. [64] with permission from Wiley, copyright 2008.

The chemical recycling of silane-XLPE in sc media was first proposed by T. Goto et al. [64]. To obtain silane-XLPE, the authors used vinyltrimethoxysilane as a crosslinker and dibutyltin dilaurate as a catalyst; after the crosslinking procedure, the gel fraction of the obtained silane-XLPE was 60%. Then silane-XLPE was decomposed in sc water and sc methanol, and the molecular weight and gel fraction were determined for the product after this treatment. The results show that when silane-XLPE is processed in sc methanol, the gel fraction is 0 at a treatment temperature above 300 °C, while for the treatment in SCW, the gel fraction is absent only at treatment temperatures above 370 °C. At the same time, during the destruction of silane-XLPE in SCW, the molecular weight of the product decreases compared to the molecular weight of the initial non-crosslinked PE, which indicates the destruction of the PE backbone as well. In sc methanol, the cleavage of crosslinking elements proceeds selectively, without degradation of the PE backbone. Analysis of the degradation products by NMR spectroscopy showed that a small amount of dimers was present in the product, but most of the siloxane crosslinks decomposed to alkoxysilane after exposure to sc methanol (Figure 5). In conclusion, the authors demonstrate that the product of chemical recycling of silane-XPE in sc methanol can be crosslinked again after re-molding, albeit with some loss of performance. In Ref. [65] the authors continue the study of the process of selective cleavage of crosslink elements in silane-XLPE, in which the results are compared when the process is carried out in various sc alcohols. It is shown that the efficiency of crosslink breaking decreases in the series: methanol, ethanol, 1-propanol, isopropanol.

After the first works demonstrated the high potential of the process under consideration, a number of further studies were focused on its technological implementation. The authors of Refs. [66,67,68] present various options for implementing a continuous process using extrusion technologies. The study [67] shows the possibility of efficient processing of silane-XLPE in sc 1-propanol on a pilot industrial plant, consisting of two extruders and providing a processing volume of 20 kg/h. In Ref. [66], processing was carried out on a model single-screw extruder in order to increase the economic efficiency of the process.

Baek and co-workers studied the kinetics of the selective de-crosslinking of silane-XLPE in sc alcohols in a batch process [69] and in a continuous process [68,70,71]. For example, in their Ref. [69], the authors described the study on the rate of de-crosslinking of silane-XLPE in a batch process in an excess of sc methanol in the temperature range of 300–400 °C. The results show that the kinetics of the process correspond to a first-order reaction with respect to the content of the gel fraction. As the temperature increases, the rate constant increases from 0.02 min^−1^ at 300 °C to 5.3 min^−1^ at 400 °C. At the same time, at temperatures above 360 °C, a decrease in the molecular weight of PE occurs. These results are of undoubted interest for an adequate choice of the optimal process parameters for the chemical recycling of silane-XLPE. The work also shows that the de-crosslinking of XLPE in sc methanol occurs more efficiently for silane crosslinks than for peroxide ones. The reaction kinetics does not undergo significant changes upon transition from a batch process to a continuous process [70], indeed, the experimental data still indicates a first-order reaction. In addition, it was confirmed that the rate constant increases with increasing temperature according to the Arrhenius law. Finally, in Ref. [71], a comparison of the kinetics of the de-crosslinking of silane-XLPE in various sc alcohols and in SCW is given. It is shown that at 380 °C the reaction rate constants for sc methanol, ethanol, 2-propanol, and water are, respectively, 2.8 min^−1^, 2.6 min^−1^, 2.4 min^−1^ and 2.1 min^−1^. The authors conclude that sc ethanol, which exhibits a reaction rate similar to sc methanol, but is not toxic, may be the optimal choice of sc alcohol for the industrial implementation of chemical recycling of silane-XLPE.

Thus, selective de-crosslinking of silane-XLPE in SCW and sc alcohols is an important example of a process, where the areas of plastic recycling in supercritical fluids and chemical recycling of silicones overlap. It should be highlighted that the efficiency of the process under consideration is directly linked to unique properties of the mentioned supercritical media. More specifically, the decrease in the polarity of water and alcohols in the sc state allows for higher affinity of the fluids towards the PE backbone, while high reactivity promotes selective de-crosslinking.

## 4. The Prospect of Chemical Recycling of Polysiloxanes in sc CO_2_

The critical pressure of sc CO_2_ is ≈74 bar, which is much lower than the critical pressure of water but comparable to the values for ethanol or propanol. The critical temperature of CO_2_ (≈31 °C) is significantly lower than that of all the sc fluids considered above, which makes it possible to carry out various chemical or technological processes in sc CO_2_ under “softer” conditions, as compared to sc water and even to sc alcohols. Supercritical CO_2_ also compares favorably with sc alcohols in that it is a non-flammable and non-toxic solvent. In addition, the fundamental difference between sc CO_2_ and SCW or sc alcohols is that under normal conditions CO_2_ is a gas: this greatly facilitates the process of its purification and reuse in industrial processes.

For the process of chemical recycling of plastics, sc CO_2_ is considered mostly as a solvent and not as an active medium, unlike SCW and sc alcohols; this is due to the high chemical inertness of CO_2_ molecules. For example, the extraction processes in sc CO_2_ are well developed from a technological point of view, which makes it possible to use sc CO_2_ to extract various functional additives from PE and PP [72,73]. Such extractions can be used for pre-treatment of plastic before its mechanical or chemical processing or for the extraction and reuse of the functional additives themselves, i.e., flame retardants, antioxidants, and others. As applied to chemical recycling, extraction with sc CO_2_ can be used to purify and separate the products of thermal oxidation of polyolefins [74]. Finally, the authors of Refs. [75,76] show that the thermochemical degradation of polyolefins in oxygen-enriched sc CO_2_ leads to the formation of paraffins and low-molecular-weight acids in high yields. The papers compare thermochemical degradation in pure oxygen under pressure and sc CO_2_ with the same amount of oxygen and show that sc CO_2_ facilitates the formation of oligomers from high-molecular-weight polyolefin molecules. The detected increase in the reaction rate of thermochemical degradation of polyolefins in sc CO_2_ can be associated with the plasticization of the polymers, which facilitates the access of oxygen molecules to polymer chains.

The foaming of plastics in sc CO_2_ can also be considered promising as part of the recycling strategy. In Refs. [77,78], the authors describe the process to obtain a polymer composite based on microcellular PP foamed in sc CO_2_ and rubber waste. Note that plastic waste can be used both as a filler, as in Refs. [77,78], and as a matrix. For example, the production of foamed PET can be a cost-effective alternative to the standard process of its mechanical or chemical recycling [79].

Polysiloxanes are a rare example of macromolecular compounds with relatively high solubility in sc CO_2_. The authors of Ref. [80] provide data on the phase behavior of PDMS of various molecular weights in sc CO_2_: at a polymer concentration of 5 wt.%, PDMS with Mw = 38,900 g/mol showed the minimum cloud point pressure at 270 bar and 47 °C; as M_w_ increases these values also increase and for PDMS with a molecular weight of 273,500 g/mol they were 380 bar and 62 °C. The authors report that further increase in M_w_ had no significant effect on the phase behavior of the system. The solubility of PDMS increases significantly with the decrease in molecular weight, and oligomers with molecular weight around 400 g/mol are soluble even in the liquid phase of two-phase CO_2_ at room temperature; at 40 °C, the minimum cloud point pressure for these oligomers in sc CO_2_ is below 100 bar in the concentration range of 1–4 wt.% [81]. The authors note that for PDMS oligomers, the structure of the end groups has a significant effect on the solubility in sc CO_2_. Low-molecular-weight organosilicon compounds, such as alkoxysilanes of various structures [82,83], chlorosilanes [84], allylsilanes, etc., are also readily soluble in sc CO_2_.

The variety of functional properties of organosilicon compounds and the unique features of sc CO_2_ as a solvent make their combination in various chemical processes highly promising. In the review paper [85], the examples of such processes are given, namely, the synthesis of polymers and polymer composites, the design of polymer membranes, including composite membranes with organosilicon or silica inclusions [86,87], as well as the modification of various surfaces with functional organosilicon compounds. From our point of view, the process of one-step synthesis of porous organosilicon matrices is of particular interest. This concept was first presented in Ref. [88], where it was shown that during the polycondensation of alkoxysilanes in sc CO_2_ with the addition of anhydrous formic acid, a porous monolithic material (aerogel) is formed in a simple one-step process that does not require time-consuming and resource-intensive stages of solvent replacement and drying. Another option for the one-step preparation of organosilicon aerogel directly in sc CO_2_ is the hydrosilylation reaction involving polymethylhydrosiloxane and PDMS oligomers with vinyl end groups [89,90]. By varying the composition and structure of macromonomers in the described process, one can effectively control the morphology and mechanical properties of the resulting porous materials [91]. The resulting aerogels are superhydrophobic, while they adsorb hydrocarbon compounds well, and thus they can be used in the field of environmental protection, namely for water purification during oil spills.

To the best of our knowledge, there are no direct examples of the use of sc CO_2_ in the chemical or mechanical recycling of polysiloxanes in the scientific literature, though the concept is very promising. Indeed, low-molecular-weight organosilicon compounds demonstrate high solubility in sc CO_2_. This fluid efficiently extracts low-molecular-weight products from polymer matrices. The polymerization reaction of silicones is an equilibrium one. Efficient extraction of low-molecular-weight cycles should pronouncedly shift the reaction towards the breakdown of polymer chains. The only problem is to find proper conditions (i.e., catalysts) for accelerating the slow reaction kinetics.

Nevertheless, the given examples of extraction and foaming in sc CO_2_ as part of recycling strategies can be fully applied to polysiloxanes. In Ref. [92] the authors demonstrate that the presence of PDMS oligomers makes it possible to increase the efficiency of PP foaming, leading to a decrease in the pore size with an increase in the uniformity of the porous structure. We believe that the detected increase in the efficiency of foaming of plastics in sc CO_2_/PDMS mixtures can be combined with the ideas outlined in Refs. [77,78]. Moreover, the use of PDMS, oligomeric and low-molecular-weight siloxanes and their derivatives as thickeners for sc CO_2_ is of particular interest for all the above mentioned recycling approaches that utilize sc CO_2_ [93]. The use of sc CO_2_ in the processes of thermal degradation of polysiloxanes also seems promising: it can be expected that an increase in the mobility of high-molecular-weight PDMS chains and a high solubility of the resulting low-molecular-weight cyclic siloxanes might lead to an increase in the efficiency of this process in sc CO_2_. Nevertheless, it must be concluded that only further scientific research can make it possible to assess the real technological prospects for the use of sc CO_2_ in the processes of the chemical recycling of polysiloxanes.

## 5. Conclusions

The thermal degradation of polysiloxanes is a well-studied process that is very promising from the point of view of chemical recycling of silicone-based wastes. Currently, research is focused on the search for new catalytic systems that can improve the efficiency of the depolymerization of polysiloxanes, and significant progress has been made in this direction. It has been demonstrated in the scientific literature that cyclic siloxanes formed during the thermal decomposition of polysiloxane wastes can be used as monomers to further production of silicone materials. Approaches to the recycling of industrial plastics in supercritical media, mainly in water and alcohols, are also being actively developed. For the plastics produced in a polycondensation process, such as PET, polycarbonates, aramids, etc., chemical recycling leads to the formation of corresponding monomers in high yields. For commonly used vinyl polymers, such as polyolefins, PVC, and PS, the product of chemical decomposition in sc water and sc alcohols consists of gas, liquid, and solid phases with broad distribution of components in each phase. Research on plastic recycling in sc water and sc alcohols is focused mainly on the most common widely used synthetic organic polymers and does not address the problems of polysiloxanes recycling. However, both in the field of silicone waste recycling and in the field of plastics recycling in supercritical media, special attention is paid to composite and crosslinked systems. In that regard, an interesting overlap in the two areas of research is the recycling of silane-crosslinked PE: the decrease in the polarity of water and alcohols during the transition to the supercritical state allows these solvents to effectively dissolve in the silane-crosslinked PE and promote the breaking of silane crosslinks. If the solvent and conditions are selected properly, this de-crosslinking occurs selectively, without degradation of the PE backbone. With regard to the recycling of high-molecular-weight silicone-based materials (both crosslinked and non-crosslinked), the prospect of using supercritical fluids to carry out this process has not yet been extensively explored in scientific studies. Meanwhile, the combination of the processes of polysiloxane depolymerization and plastic recycling in sc media that were considered in the present review can potentially lead to the emergence of a synergistic effect necessary for a breakthrough in the real industrial applications of both the chemical recycling of siloxanes and supercritical fluid technologies.

## Data Availability

The data presented in this study are available on request from the corresponding author.
